# An Ionic Liquid Supramolecular Gel Electrolyte with Unique Wide Operating Temperature Range Properties for Zinc-Ion Batteries

**DOI:** 10.3390/polym16121680

**Published:** 2024-06-13

**Authors:** Hui Li, Changmiao Huang, Zixuan Teng, Yushu Luo, Chaocan Zhang, Lili Wu, Wenchao Huang, Tingting Zhao, Lijie Dong, Wanyu Chen

**Affiliations:** School of Materials Science and Engineering, Wuhan University of Technology, Wuhan 430070, China; lh370765288@whut.edu.cn (H.L.); 317428@whut.edu.cn (C.H.); 281463@whut.edu.cn (Z.T.); 281387@whut.edu.cn (Y.L.); polymers@whut.edu.cn (C.Z.); polym_wl@whut.edu.cn (L.W.); wenchao.huang@whut.edu.cn (W.H.); zhaotingting@whut.edu.cn (T.Z.); dong@whut.edu.cn (L.D.)

**Keywords:** supramolecular gel electrolyte, gelator, zinc-ion battery, ionic liquid

## Abstract

Zinc-ion batteries are promising candidates for large-scale energy storage. The side reactions of the hydrogen evolution reaction (HER) and zinc dendrite growth are major challenges for developing high-performance zinc-ion batteries. In this paper, a supramolecular gel electrolyte (BLO-ILZE) was self-assembled in an ionic liquid (EMIMBF_4_) with zinc tetrafluoroborate (Zn(BF_4_)_2_) on the separator in situ to obtain a gel electrolyte used in zinc-ion batteries. BLO-ILZE is demonstrated to significantly enhance conductivity over a broad temperature range between −70 and 100 °C. Interestingly, through testing and fitting, it is found that the supramolecular gel electrolyte satisfies the liquid state law over a wide temperature range, and even achieves high conductivity (2.12 mS cm^−1^) at −40 °C. It is equivalent to the conductivity of aqueous zinc-ion batteries (ZnSO_4_/H_2_O) at −10 °C, which is 2.33 mS cm^−1^. Moreover, the supramolecular gel electrolyte can effectively inhibit the HER, thus exhibiting a longer lifetime in Zn/Zn cells for 3470 h at 1 mA cm^−2^ compared to the aqueous zinc-ion batteries with the Zn(BF_4_)_2_ aqueous electrolyte (400 h at 1 mA cm^−2^). The assembled V_2_O_5_/BLO-ILZE/Zn full cells also showed cycling performance, with 5000 cycles at 0.5 mA g^−1^ at room temperature, a capacity of 98%, and a coulombic efficiency of about 100%.

## 1. Introduction

With the decreasing fossil fuel reserves and increasing environmental awareness [1], the exploration of green and sustainable energy resources has increased [2]. At present, zinc-ion batteries (ZIBs) are receiving extensive attention owing to their high abundance [3], intrinsic safety, high theoretical capacity, suitable electrochemical potential (−0.762 V vs. standard hydrogen electrode), and low cost [4,5]. Hence, because of these advantages, aqueous zinc-ion batteries have been considered promising alternative energy storage devices [6,7,8,9,10].

However, since the interphase between the Zn anode and the aqueous electrolyte still faces severe inherent problems [11], the Zn anode is thermodynamically unstable in an aqueous system [12]. Manickam Minakshi et al. [13,14] used FePO_4_ as a battery cathode material in aqueous lithium hydroxide (LiOH), which formed intercalated LiFePO_4_ reversibly on electroreduction/oxidation to solve these problems. Some key issues, including metal corrosion, complex side reactions, and uncontrollable Zn dendrite caused by Zn metal anode redox chemistry, still hinder the practical application of zinc-ion batteries (ZIBs) [15,16,17,18,19]. Water molecules in aqueous electrolytes often decompose during the charging/discharging process, accompanied by H_2_ or O_2_ evolution and the formation of byproducts, such as ZnO, Zn(OH)_2_, and [Zn(OH)_4_]^2−^). Their poor anti-freezing/heat-resistant properties at high and low temperatures pose another challenge for their practical application in aqueous ZIBs [20].

Ionic liquids (ILs) are potential alternatives to aqueous electrolytes, providing several advantages such as low flammability, low volatility, and most crucially, high (electro) chemical stability [21,22,23]. ILs may provide the possibility of increasing cycling stability and enhancing battery safety. They can enable the battery to have a wide operating temperature range [24,25,26]. Currently, ILs are used as electrolytes for Li-ion batteries due to their cost, safety, and performance. However, the use of ILs as solvents in Zn-ion batteries has rarely been studied, and most of them are used as electrolyte additives for aqueous ZIBs to inhibit the side reactions and Zn dendrite growth during the Zn plating process. For example, Liu, Z et al. [23] used an ionic liquid choline acetate with 30 wt% of water as an electrolyte for zinc-ion batteries; the Zn/PBA cell exhibited a specific capacity of about 120 mAh g^−1^ at a current of 10 mA g^−1^ (similar to 0.1 C), and dendrite-free Zn deposits were obtained after 100 deposition/dissolution cycles.

In addition, it is important to improve the low-temperature performance for practical application of zinc-ion batteries. At present, the most commonly used method is to add an anti-freeze additive or corrosion inhibitor to the electrolyte and to improve the electrode material to increase the range of temperature. Thuy Nguyen Thanh Tran et al. [27] modified the electrolyte in aqueous zinc-ion batteries (1 M ZnSO_4_) by adding suitable amounts of ethylene glycol to improve the anti-freezing ability of the aqueous electrolyte and increase the conductivity of electrolyte at low temperatures. 

Gel electrolytes combine the advantages of liquid and solid components, which can confine water and avoid the risk of leakage. Hence, they exhibit a promising application prospect in ZIBs. Tingting Wei et al. [28] reported a hydrogel electrolyte composed of polyacrylamide (PAM), ZnSO_4_ (ZS), glycerol (GL), and acetonitrile (AN) (denoted as ZS/GL/AN) for an aqueous zinc-ion battery with excellent mechanical properties, high ionic conductivity, and wide operating temperature range (−20~60 °C). The batteries showed excellent cycling properties and capacity performance (138 mAh⋅g^−1^ at −20 °C and 262 mAh⋅g^−1^ at 60 °C). However, most of the current gel electrolytes are chemically crosslinked, which needs to be improved in terms of easy operation, high conductivity, wide temperature range, and long cycle stability.

Supramolecular gels are novel kinds of soft materials assembled by a gelator via non-covalent interactions such as hydrogen bonds, metal-to-ligand coordination, and electrostatic or host–guest interactions [29]. Supramolecular gel electrolytes can actively reconfigure themselves with the ability to remodel, reshape, and adapt to environmental conditions [30]. Supramolecular materials have potential applications in ZIB electrolytes due to their biocompatibility, controllable self-assembly, and environmental friendliness. Currently, research is focused on their application in the modification of zinc cathode interfaces or as electrolyte additives [31]. And studies on supramolecular gel electrolytes for ionic liquid zinc-ion batteries are still very few and unsystematic. 

In this study, a gelator was self-assembled on a separator in situ to form a usable supramolecular gel electrolyte in an ionic liquid for zinc-ion batteries. The most significant advantages of the system were reduced dendrite Zn plating/stripping, decreased side reactions of the hydrogen evolution reaction, a wide temperature window (−70~100 °C), and easy operation. The 1-ethyl-3-methylimidazolium tetrafluoroborate ([EMIM]BF_4_) ionic liquid with zinc tetrafluoroborate (Zn(BF_4_)_2_) was prepared as an electrolyte (denoted as ILZE). Then, we used the gelation of the ILZE by a novel oxalamide gelator for Zn-ion batteries (denoted as BLO-ILZE). The BLO-ILZE electrolyte (5 wt% BLO gelator) prevented the occurrence of hydrogen evolution in aqueous systems. And it restrained the growth of zinc dendrite on the electrode. The symmetric Zn/Zn cell with the BLO-ILZE electrolyte had better cycling stability than the ILZE and the Zn(BF_4_)_2_ aqueous electrolyte, indicating that the inhibition effect on zinc dendrite was remarkable. SEM, EDS, XRD, and laser microscopy were used to test and analyze the reasons for the stability improvement, demonstrating that the cycle time of symmetrical cells with supramolecular gel electrolyte was 8.5 times higher than that of aqueous zinc-ion batteries.

## 2. Experimental Section

### 2.1. Materials

Toluene (AR, 90%) and Acetone (AR, 90%) were purchased from Wenzhou Dingcheng Chemical Co., Ltd. (Wenzhou, China). Zinc fluoborate hydrate (Zn(BF_4_)_2_·xH_2_O, Zn ≥ 18%), 1-Ethyl-3-methylimidazolium tetrafluoroborate (EMIMBF_4_, anhydrous, 98%), L-Leucinol (anhydrous, 96%), Diethyl oxalate (anhydrous, 99%), Isopropyl ether (anhydrous, >98.0%), and Ethanol (anhydrous, ≥99.5%, moisture ≤0.005%) were purchased from Aladdin (Shanghai, China). Vanadium pentoxide commercial electrode (V_2_O_5_, 10 μm), Fiberglass diaphragm (GF/D), and Button battery case (CR2032) were provided by Guangdong Canrd New Energy Technology Co., Ltd. (Guangdong, China). Copper sheets (99.99%) and Zinc metal sheets (99.99%) were purchased from Xinyi Metal Co., Ltd. (Xingtai, China).

### 2.2. Synthesis of BLO Gelator

The BLO gelator was prepared according to the reported synthetic route [32,33]. L-Leucinol (4 mmol) was suspended in toluene (10–20 mL) and diethyl oxalate (2 mmol) was added as the solution was vigorously stirred. The reaction mixture was stirred and heated by reflux at 90 °C for 1 h. The initially formed solution quickly transformed into a dense gelatinous mass. After cooling to room temperature, the mixture was diluted with isopropyl ether (10–20 mL) until the gelatinous mass was destroyed, and the crystalline product was separated by filtration. The BLO gelator was obtained.

### 2.3. Preparation of BLO-ILZE Electrolyte

**Figure 1:**
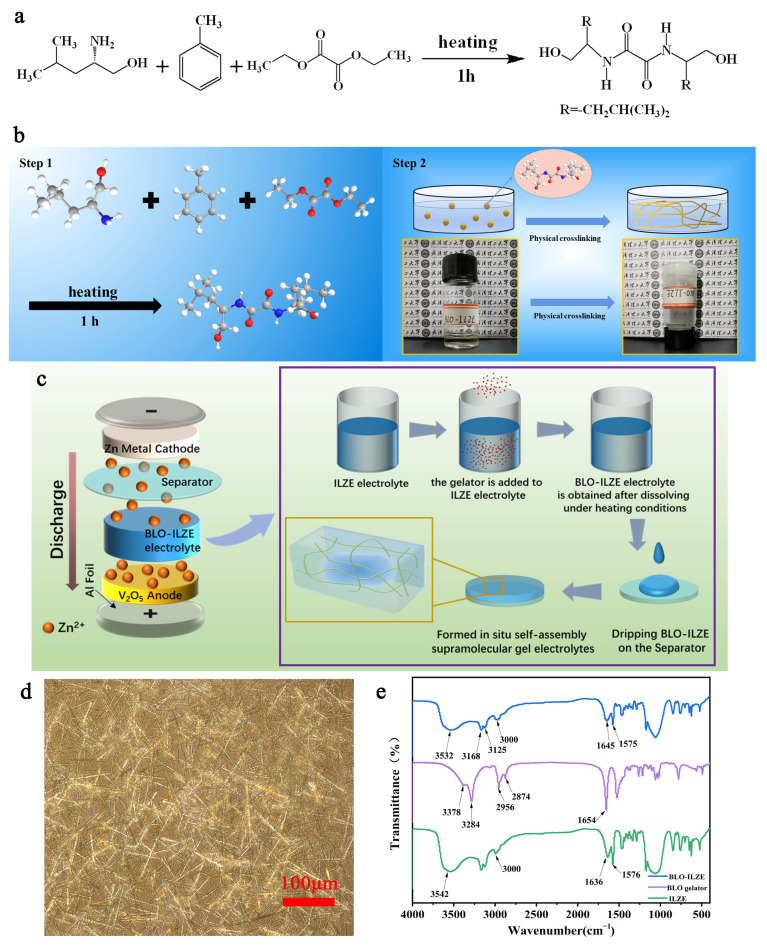
(**a**) The chemical equation of BLO gelator preparation. (**b**) Schematic diagram of the preparation of BLO-ILZE electrolyte and the structure of BLO gelator (the background is Wuhan University of Technology). (**c**) Preparation of BLO-ILZE electrolyte. (**d**) Optical micrograph of the BLO-ILZE gel electrolyte. (**e**) The infrared spectra of the BLO gelator, BLO-ILZE electrolyte and the ILZE electrolyte.

The 1.434 g of Zn(BF_4_)_2_ was dissolved into 3 mL EMIMBF_4_ at 60 °C oil bath condition and vigorously stirred for 2 h [34]. The transparent ionic liquid-based Zn salt electrolyte (ILZE) was achieved, after the solution was cooled down at room temperature condition. The prepared ILZE electrolyte was added to a glass bottle with a concentration of 5 wt% BLO gel factor and heated at 90 °C for 1 h in a water bath. After the BLO galetor was completely dissolved in the solution, the solution was used as a stock solution. Afterwards, 120 mL of the stock solution was quickly taken and added dropwise to a glass fiber (GF) membrane (F = 16 mm). When the temperature decreased to the gelation temperature, the BLO gelator began to self-assemble in ILZE, and after the solution was cooled down under room temperature conditions, the gel electrolyte was self-assembled in situ on the GF membrane. The procedure for preparing BLO-ILZE electrolyte is shown in Figure 1b,c.

### 2.4. Characterization

The coin-cells were discharged/charged at 1 mA g^−1^ and stopped at certain potentials for 60 min, and then disassembled. The electrodes were washed with deionized water several times and then dried in vacuum. The morphology of the electrodes was analyzed by X-ray diffraction (XRD, D8 Advance X, Bruker, AXS, Karlsruhe, Germany), using Cu-Kα as the target, with a scanning range of 20–100° and a scanning speed of 10 mv/s.

The microstructure, morphology, and atomic composition of the electrolyte were characterized by using a field emission scanning electron microscope with an X-Max N80 spectrometer (FE-SEM, JSM-7500F, JEOL, Tokyo, Japan).

The composition of the electrolyte was measured by using an Intelligent Fourier Transform Infrared Spectrometer (Nexus, Thermo Nicolet Corporation, Madison, WI, USA) with a wavelength range of 4000–400 nm.

Polarizing light microscopy-CX40P (Sunny Opticaltechnology (Group) Co., Ltd., Ningbo, China) was used to characterize the morphology of gel electrolytes.

The thermodynamic properties of supramolecular gel electrolytes and other liquid electrolytes were measured by a differential scanning calorimeter (TA-DSC2500, American TA Instruments, New Castle, DE, USA) at 10 °C/min with a scanning temperature zone of −80~200 °C.

An electrochemical workstation (CHI660, Shanghai Chenhua Instrument Co., Ltd., Shanghai, China) was used to measure the electrochemical impedance spectroscopy (EIS) behavior of the electrolyte at a frequency of 0.01 Hz–0.1 MHz and a temperature of −70~100 °C. The structure of the test device was a conductance electrode (DJS-1VTC, INESA Scientific Instrument Co., Ltd., Suzhou, China). The two electrode plates of the conductive electrode are pt, and the middle distance between the two electrode plates is a fixed value. The equivalent circuit diagram of the electrolyte was obtained by fitting using the Zview software package (Zview version:2.70).

The NEWARE Battery tester (CT-4008-5 V20 mA-164, Shenzhen NEWARE Electronics Co., Ltd., Shenzhen, China) was used to test the constant current charge and discharge of button batteries at a temperature of 24 °C. The cell type used was a button battery.

### 2.5. Binding Energy Calculations

All structures were optimized by DFT using generalized gradient approximation with the Perdew–Burke–Ernzerhof functional. The binding energies Eb between two samples were calculated as:

(1) $$E_{b}\left({Zn}^{2+}-{BF}_{4}^{-}\right)=E\left({Zn}^{2+}-{BF}_{4}^{-}\right)-E\left({Zn}^{2+}\right)-E\left({BF}_{4}^{-}\right)$$

(2) $$E_{b}\left({Zn}^{2+}-H_{2}O\right)=E\left({Zn}^{2+}-H_{2}O\right)-E\left({Zn}^{2+}\right)-E\left(H_{2}O\right)$$

(3) $$E_{b}\left({Zn}^{2+}-BLO\right)=E\left({Zn}^{2+}-BLO\right)-E\left({Zn}^{2+}\right)-E\left(BLO\right)$$

(4) $$E_{b}\left({EMIM}^{+}-{BF}_{4}^{-}\right)=E\left({EMIM}^{+}-{BF}_{4}^{-}\right)-E\left({EMIM}^{+}\right)-E\left({BF}_{4}^{-}\right)$$

(5) $$E_{b}\left({EMIM}^{+}-H_{2}O\right)=E\left({EMIM}^{+}-H_{2}O\right)-E\left({EMIM}^{+}\right)-E\left(H_{2}O\right)$$

(6) $$E_{b}\left({EMIM}^{+}-BLO\right)=E\left({EMIM}^{+}-BLO\right)-E\left({EMIM}^{+}\right)-E\left(BLO\right)$$

(7) $$E_{b}\left(BLO-{BF}_{4}^{-}\right)=E\left(BLO-{BF}_{4}^{-}\right)-E\left(BLO\right)-E\left({BF}_{4}^{-}\right)$$

(8) $$E_{b}\left(BLO-H_{2}O\right)=E\left(BLO-H_{2}O\right)-E\left(BLO\right)-E\left(H_{2}O\right)$$

(9) $$E_{b}\left(H_{2}O-{BF}_{4}^{-}\right)=E\left(H_{2}O-{BF}_{4}^{-}\right)-E\left(H_{2}O\right)-E\left({BF}_{4}^{-}\right)$$

### 2.6. Electrochemical Measurements

Ionic conductivity was determined by electrochemical impedance spectroscopy (EIS) with the aid of electrochemical workstation (CHI660, Shanghai Chenhua Instrument Co., Ltd., Shanghai, China) at the temperature range of 25 °C. The ILZE and BLO-ILZE electrolytes were dropped into test tubes, which were sandwiched between two symmetrical platinum black (Pt) electrodes (Pt/electrolyte/Pt). Ionic conductivity was calculated by

(10) $$\sigma =\frac{l}{RS}$$

where *l* (cm) was the distance between platinum black electrodes, *S* was the area contacting with the Pt, and *R* (Ω) was the impedance measured by EIS. Here *l*/*S* was a fixed value of 2.

## 3. Results and Discussion

### 3.1. Preparation of BLO-ILZE Electrolyte

The gelator is synthesized from L-leucinol, toluene, and diethyl oxalate (Figure 1a) [32,33]. As shown in Figure 1b, the gelator is added to the ILZE electrolyte and self-assembled to become BLO-ILZE electrolyte. The preparation of the BLO-ILZE electrolyte is shown in Figure 1c. This physically cross-linked supramolecule gel electrolyte can be self-assembled on the separator in situ to directly obtain usable gel electrolyte. Typically, chemically cross-linked gel electrolytes require gel synthesis, and then sections are immersed in the electrolyte until swelling equilibrium is achieved [35]. The gelator self-assembled to 3D networks to wrap ILZE in the network (Figure 1d). From a macroscopic point of view, the liquid state changes to a gel state, and it is in a non-flowing state after being inverted. The supporting Information shows a multimedia file about the process of self-assembly of BLO galetor in ILZE photographed under a microscope. The fibers gradually grew from nothing and intertwined with each other to form a 3D network structure.

The characterization of the ILZE electrolyte, BLO gelator, and BLO-ILZE electrolyte are investigated by Fourier transform infrared spectroscopy (FT-IR), as shown in Figure 1e. Tables S1 and S2 show the positions corresponding to different peaks of ILZE electrolyte and BLO gelator (see Supporting Information). The BLO gelator will self-assemble to form BLO-ILZE when added to ILZE. For BLO-ILZE, the peak at 3532 cm^−1^ is ascribed to the O-H stretching vibrations of the intramolecular hydrogen bonding of ILZE. The peaks at about 3168 cm^−1^ and 3125 cm^−1^ are attributed to asymmetric stretching vibrations and symmetric stretching vibrations of the N-H groups on the amides of the BLO gelator. The peak at 3000 cm^−1^ is ascribed to the -C=C-H stretching vibration peak of ILZE. The peak at 1645 cm^−1^ was ascribed to the C=O stretching vibration of ILZE and BLO gelator. The C=O peak was blue-shifted and shifted towards a lower wave number. The peak at 1575 cm^−1^ may be due to C=N^+^ stretching vibration of the ILZE [36]. It indicated that the gel is formed by hydrogen bonds between L-leucosine and diethyl oxalate. Because 5 wt% BLO gelator was added to ILZE, the concentration of ILZE was large, so the peaks of BLO-ILZE were mostly similar to that of ILZE. All the above suggest that the gel electrolyte is formed by hydrogen bonds between the ILZE and the BLO gelator.

### 3.2. The Conductivity of the Electrolytes

**Figure 2:**
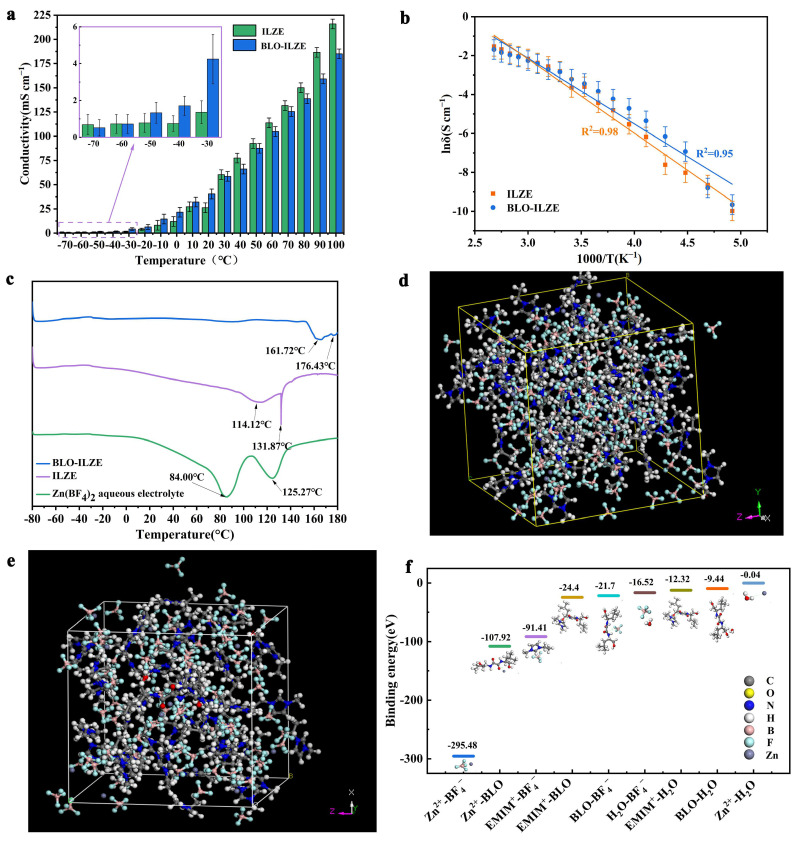
(**a**) The ionic conductivities of ILZE and BLO-ILZE electrolytes. (**b**) The ionic conductivity–temperature curve of the ILZE and BLO-ILZE fitted by the Arrhenius equation. (**c**) DSC diagram of the Zn(BF_4_)_2_ aqueous electrolyte, ILZE electrolyte, and BLO-ILZE electrolyte. (**d**) Snapshot of a typical MD simulated cell for ILZE electrolyte. (**e**) Snapshot of a typical MD simulated cell for BLO-ILZE electrolyte. (**f**) Relative binding energies between any two ingredients in the Zn(BF_4_)_2_ aqueous electrolyte, ILZE electrolyte, and BLO-ILZE electrolyte obtained from DFT calculations.

Electrochemical impedance spectroscopy (EIS) diagrams are used to analyze the ionic conductivity. Figures S1 and S2 in the supporting Information show electrochemical impedance plots of the ILZE electrolyte and BLO-ILZE electrolyte at different temperatures and their equivalent circuit diagrams fitted with the Zview software package. Figure S3 shows the Electrochemical impedance plots of 2M Zn(BF_4_)_2_, the ILZE electrolyte, and different concentrations of the BLO-ILZE supramolecular gel electrolyte at 25 °C. It can be seen that the resistance of the BLO-ILZE5 (5 wt%) supramolecular gel electrolyte at room temperature is very close to that of 2M Zn(BF_4_)_2_ and the ILZE electrolyte. However, the BLO-ILZE7.5 (7.5 wt%) and BLO-ILZE10 (10 wt%) supramolecular gel electrolytes are more resistive in comparison and may affect Zn^2+^ transport, thereby affecting the battery performance. Figure 2a,b both show the ionic conductivity. Figure 2a shows the ionic conductivities of ILZE and BLO-ILZE electrolytes. Figure 2b shows the ionic conductivity–temperature curve of the ILZE and BLO-ILZE fitted by the Arrhenius equation. The ionic conductivities of the ILZE and BLO-ILZE electrolytes at different temperatures are shown in Figure 2a. At 20 °C, the conductivities of ILZE and BLO-ILZE are 26.26 mS cm^−1^ and 40.40 mS cm^−1^, respectively. At −10 °C, the conductivities of ILZE and BLO-ILZE are 8.13 mS cm^−1^ and 14.60 mS cm^−1^, respectively. And their conductivities are significantly better than that of ZnSO_4_, which is 2.33 mS cm^−1^ at −10 °C [37,38,39]. In the electrolytes of the water system, when the temperature is lower than −10° C, the ionic conductivity of the ZnSO_4_ liquid electrolyte is too small to measure due to the freezing of the electrolyte [40]. But the conductivities of ILZE and BLO-ILZE electrolytes can still be measured. At −60 °C, the conductivities of ILZE and BLO-ILZE are 0.17 mS cm^−1^ and 0.15 mS cm^−1^, respectively. The conductivities of BLO-ILZE electrolyte and ILZE are divided by −60 °C. At temperatures above −60 °C, the conductivity of BLO-ILZE electrolyte is lower than that of ILZE, but the difference is not significant. At −40 °C, the conductivities of ILZE and BLO-ILZE are 0.49 mS cm^−1^ and 2.12 mS cm^−1^, respectively. A differential scanning calorimeter (DSC) is used to study the thermodynamic properties of electrolytes (Figure 2c and Figure S4). Scanning from 0 °C to −80 °C, no significant freezing point is seen in all three electrolytes. This indicates that ILZE and BLO-ILZE still have good electrical conductivities at −40 °C. At a temperature of 30 °C, the conductivity of the BLO-ILZE electrolyte and the conductivity of ILZE are basically the same. Above 30 °C, the conductivity of ILZE increases with increasing temperature, exceeding the conductivity of BLO-ILZE. At 90 °C, the conductivities of ILZE and BLO-ILZE are 186.57 mS cm^−1^ and 159.24 mS cm^−1^, respectively. At 100 °C, the conductivities of ILZE and BLO-ILZE are 215.96 mS cm^−1^ and 185.01 mS cm^−1^, respectively. Because the boiling point of water is 100 °C, the aqueous zinc-ion batteries are extremely unstable at high temperatures and cannot be used. As shown in Figure 2c, scanning from −80 °C to 180 °C, the decomposition temperatures of ILZE and BLO-ILZE electrolytes are 114.12 °C and 161.72 °C, respectively. The Zn(BF_4_)_2_ liquid aqueous electrolyte is already unstable at 84 °C. It further explains that ILZE and BLO-ILZE electrolytes have wide temperature windows. Figure S4 in the Supporting Information shows a DSC diagram of 2M Zn(BF_4_)_2_, EMIMBF_4_, and ILZE. It can be observed that the freezing point of EMIMBF_4_ ionic liquid is −46.22 °C. Although the freezing point of the ILZE electrolyte cannot be observed, as the content of EMIMBF_4_ ionic liquid in ILZE electrolyte is extremely high, this can well explain the sudden decrease in the conductivity of ILZE electrolyte at −40 °C. The freezing point of BLO ILZE is below this temperature. This explains the reason about the conductivity of BLO-ILZE is higher than that of ILZE at −40 °C. It shows that the three-dimensional network of supramolecular gels has almost negligible hindrance to Zn^2+^ migration. The addition of ionic liquid and the gelation of electrolyte can increase the temperature range of electrolytes.

As shown in Figure 2b, the relationship between ionic conductivity and temperature of supramolecular gel electrolyte is calculated by fitting by Arrhenius formula:

(11) $$\delta =Aexp\frac{-E\alpha }{KT}$$

Here, δ is the ion conductivity, *K* is the rate constant, *Eα* is the apparent activation energy, A is the frequency factor, and *T* is the thermodynamic temperature. It can be seen from Figure 2b that the curve fitted with the Arrhenius formula and that the linearity was decent at high temperature and low temperature due to a wide temperature window of the electrolyte. The R^2^ obtained by fitting is 0.98 for ILZE and 0.95 for BLO-ILZE. The fitted cases of ILZE and BLO-ILZE electrolytes are close to liquid, following the liquid state mechanism. This indicates that ILZE and BLO-ILZE electrolytes can operate within a wide temperature window −70~100 °C. And as shown in Figure 2c, the DSC results scanning from approximately −80 to 180 °C provide a further evidence for the reason why ILZE and BLO-ILZE electrolytes still have regular linear fitting phenomena in the low temperature region. It shows that ILZE and BLO-ILZE electrolytes can be used in extreme environments.

To further study the mechanism, density functional theory (DFT) calculations and molecular dynamics (MD) simulations are used to verify the interactions within the electrolyte (Figure 2d–f). DFT calculations show that the relative binding energies between any two ingredients in the Zn(BF_4_)_2_ aqueous electrolyte, ILZE electrolyte, and BLO-ILZE electrolyte follow an order of Zn^2+^-BF_4_^−^ > Zn^2+^-BLO > EMIM^+^-BF_4_^−^ > EMIM^+^-BLO > BLO-BF_4_^−^ > H_2_O-BF_4_^−^ > EMIM^+^- H_2_O > BLO-H_2_O > Zn^2+^-H_2_O (Figure 2f). The results show the binding energy of EMIM^+^ and BLO is lower than that of Zn^2+^ and BLO, indicating that EMIM^+^ is more inclined to interact with BLO to form the more stable self-assembled gel networks. It is beneficial to the migration of Zn^2+^ between the 3D gel networks. In the Zn(BF_4_)_2_ aqueous electrolyte, Zn^2+^ is more inclined to interact with H_2_O, indicating that the water in the Zn(BF_4_)_2_ aqueous electrolyte will easily accumulate around Zn^2+^, leading to the formation of Zn dendrite [41]. All of the aspects mentioned above indicate that it will be beneficial to inhibit the dendrite growth and uniform Zn deposition in the BLO-ILZE gel electrolyte [42,43].

### 3.3. Electrochemical Performance of Symmetric Zn/Zn Cells

**Figure 3:**
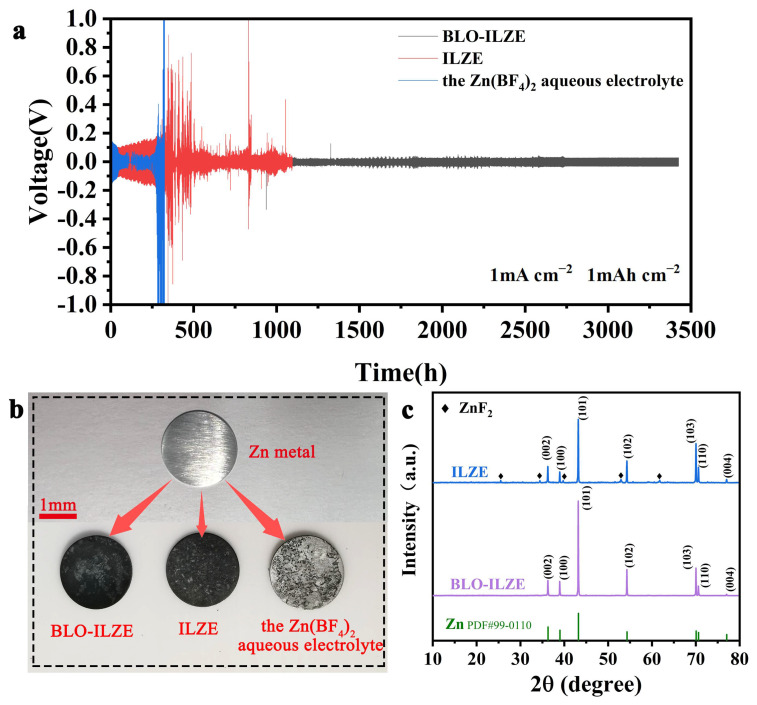
(**a**) Galvanostatic Zn plating/stripping in Zn/Zn symmetrical cells with 1 mA cm^−2^. (**b**) The optical photographs of the zinc foils after circulation. (**c**) The XRD pattern of the Zn electrode after 20 cycles at a current density of 1 mA cm^2^.

Symmetric Zn/Zn cells are employed to estimate the Zn^2+^ stripping/plating performance of the hybrid electrolyte (Figure 3a and Figure S5). As shown in Figure 3a, the symmetrical cells assembled with the BLO-ILZE electrolyte exhibited great cycling stability for nearly 3470 h at a current density of 1 mA cm^−2^ and a capacity density of 1 mA h cm^−2^. The BLO-ILZE electrolyte used in the symmetric cells displayed a much longer cycle lifespan than the Zn(BF_4_)_2_ aqueous electrolyte and ILZE electrolyte. In contrast, the symmetrical cell with the Zn(BF_4_)_2_ aqueous electrolyte could only cycle for less than 400 h and then caused a short circuit due to the uncontrollable dendrite formation and side reactions during cycling. The cycle time of symmetrical cells with the BLO-ILZE electrolyte is 8.5 times higher than that of aqueous zinc-ion batteries with the Zn(BF_4_)_2_ aqueous electrolyte. Table S3 in the Supporting Information shows the cycle time of symmetrical cells with the BLO-ILZE electrolyte is 8.6 times higher than that of aqueous zinc-ion batteries with the ZnSO_4_ aqueous electrolyte, and is 38.5 times higher than that of aqueous zinc-ion batteries with the ZnBF aqueous electrolyte. In the BLO-ILZE electrolyte, a stable thin solid electrolyte interface (SEI) layer forms on the surface of the zinc electrode. The Zn(BF_4_)_2_ aqueous electrolyte can easily pierce the separator and cause a short circuit in the battery because of the growth of Zn dendrite, thereby reducing the cycle life of the battery [44,45]. Figure S5 shows the voltage profiles of Zn/Zn symmetrical batteries with different electrolytes at the current density of 0.5 mA cm^−2^ and 5 mA cm^−2^. It can be seen that the polarization voltage of 2M Zn (BF_4_) _2_ is unstable during cycling, indicating that the transmission of Zn^2+^ is hindered during cycling. In contrast, the stability of symmetrical cells using the ILZE and BLO-ILZE electrolytes is effectively enhanced. However, the polarization voltage of the BLO-ILZE electrolyte is more stable than that of the ILZE electrolyte, probably because the 3D network limits the movement of other positive and negative ions in the ILZE electrolyte which improves the stability. Figure 3b shows an optical photograph of the zinc foils after circulation. As shown in Figure 3b, after a period of circulation in the Zn(BF_4_)_2_ aqueous electrolyte, the surface of the Zn foil is rough, and a large number of non-uniform zinc dendrites can be clearly seen. The Zn foils after a period of cycling in ILZE and BLO-ILZE electrolytes have relatively flat surfaces, and uniform SEI layers can be seen. The long-term cycling properties of BLO-ILZE electrolytes with different concentrations of BLO galetor content have also been investigated. Figure S6 shows the voltage profiles of Zn/Zn symmetrical batteries with different concentrations of the BLO-ILZE electrolyte at the current density of 0.5 mA cm^−2^, 1 mA cm^−2^, and 2 mA cm^−2^. It is found that BLO-ILZE electrolyte with a BLO content of 5 wt% has the best long-term cycling stability, and can affect the performance of the battery by adjusting the concentration of galetor to affect the 3D network structure. Therefore, 5 wt% BLO galetor is used in all subsequent studies.

An X-ray diffraction (XRD) analysis provides further evidence to verify the weak side reactions in electrolytes. XRD is carried out to characterize the zinc foils after cycling, as shown in Figure 3c. The fresh Zn metal foils are used as the positive and negative electrodes of the symmetrical cell. After 20 cycles of constant current charge and discharge, the Zn foils were collected, washed, and dried for XRD analysis. In ILZE, the sample shows a few diffraction peaks in XRD patterns that may due to the water-induced side reactions. Similarly to the results of Daliang Han et al. [46], the water-induced side reactions will generate the dense and stable ZnF_2_ layer on the surface of Zn anode. In contrast, almost no new diffraction peaks appeared after 20 cycles of constant current charge and discharge in BLO-ILZE. After 20 cycles, the ZnF_2_ stemming from the side reactions was detected on the surface of the Zn foil in ILZE electrolytes. In contrast, the XRD peaks from the ZnF_2_ are hardly noticeable because of the weak side reactions between Zn and the BLO-ILZE electrolyte.

**Figure 4:**
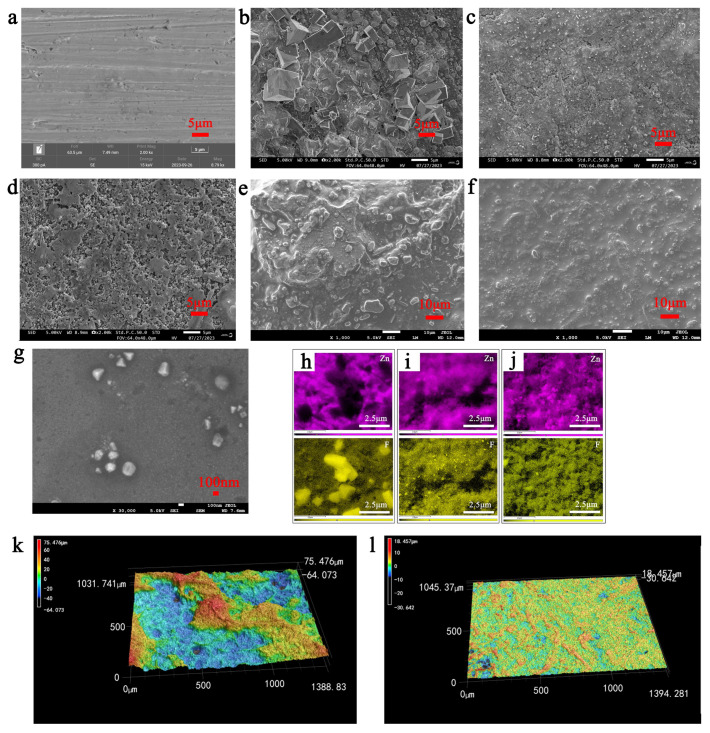
(**a**) SEM images of the initial Zn metal surface in the Zn/Zn symmetric cells. (**b**–**d**) SEM images of the Zn metal surface in the Zn/Zn symmetric cells after cycling numbers of 20 with the Zn(BF_4_)_2_ aqueous electrolyte (**b**), ILZE (**c**), and BLO-ILZE (**d**). (**e**–**g**) SEM images of the Zn metal surface in the Zn/Zn symmetric cells after 3000 cycling numbers with ILZE (**e**) and BLO-ILZE at different magnifications (**f**,**g**). (**h**) EDS images of the anode after cycled in the Zn(BF_4_)_2_ aqueous electrolyte. (**i**) EDS images of the anode after cycled in ILZE electrolyte. (**j**) EDS images of the anode after cycled in BLO-ILZE electrolyte. (**k**) The corresponding 3D surface topographies of the cycled Zn electrode in ILZE electrolyte. (**l**) The corresponding 3D surface topographies of the cycled Zn electrode in BLO-ILZE electrolyte.

In order to further find out the reason for the stability of the long cycle, the morphology of the Zn anodes after cycle are studied by scanning electron microscopy (SEM). Figure 4a–g show the morphological evolution of Zn anodes with plating time. The surfaces of Zn anodes at the initial state were smooth and flat (Figure 4a). A large number of Zn dendrites clustered on the surface of the zinc anode with the Zn(BF_4_)_2_ aqueous electrolyte. Zn dendrite accumulation pierced the diaphragm and caused short circuit, which corresponded to the results of Figure 4b. Figure 4b–d show the typical SEM images of Zn electrodes after 20 cycles in the Zn(BF_4_)_2_ aqueous electrolyte, ILZE electrolyte, and BLO-ILZE electrolyte. In the Zn(BF_4_)_2_ aqueous electrolyte, loose deposited Zn with a dendrite-like morphology is observed on the surface of the Zn electrode. More and larger zinc dendrites are found after 20 cycles. This deposition morphology of zinc due to the short circuit phenomenon of Zn/Zn symmetric batteries after dozens of cycles. Figure 4c displays the SEM image of Zn in ILZE after 20 cycles, revealing the non-uniform Zn deposition morphology. In contrast, Zn deposition is smooth and dense in the BLO-ILZE electrolyte after 20 cycles (Figure 4d). For further comparison, Figure 4e–g show SEM images of the Zn anode after 3000 cycles. Figure 4e shows the surface topography of the Zn anode after 3000 cycles in ILZE. It can be clearly seen that after a long period of cycling, a large number of by-products appear on the surface of the Zn anode, and the SEI layer of uneven height is stacked. Figure 4f shows the surface topography of the Zn anode after 3000 cycles in BLO-ILZE. In contrast to the ILZE, the surface of the Zn anode is flatter after a long cycle in BLO-ILZE. Figure 2g is a magnified view of Figure 2f. It can be seen that the surface of the Zn anode is still very flat, and there is only about 100~200 nm of dendrite formation. The results show that BLO-ILZE can inhibit the generation of dendrite and generate a uniform and dense SEI layer, thereby prolonging the service life of the battery. As reported, hydrogen evolution reaction and oxygen evolution reaction are inevitable in liquid electrolytes and a typical tip-growth theory can describe the deposition behavior of Zn^2+^ in a liquid electrolyte [47]. At the beginning of the deposition, Zn will be deposited on the substrate and unavoidably form some tips. As known, a tip on the anode surface builds a stronger electrical field, and thus, more Zn^2+^ ions prefer to accumulate and deposit around the tips rather than on smooth regions [48]. Figure S10 shows the schematic image of interfacial change at the interface of the electrode/electrolyte. In the liquid electrolyte, such Zn tips inevitably grow into vast Zn slices with bulk aggregations and non-uniform deposition in the electrolyte and finally end up cracking to form “dead-Zn” without electrochemical activity [49,50]. In comparison, after 20 cycles at 1 mA cm^−2^, the surface of the Zn anode was smoother and flatter, with no obvious irregular flakes or filament. This indicates that the ILZE and BLO-ILZE electrolytes could effectively inhibit the growth of zinc dendrite because water is replaced with an ionic liquid. In addition, it can be seen in Figure 4 that the Zn anode in BLO-ILZE has fewer Zn dendrites than in the ILZE electrolyte. 

Because the movement of the Zn^2+^ ions in BLO-ILZE electrolyte is constrained and confined by the gel electrolyte due to the pores between the gel 3D networks [51]. An optical micrograph of the BLO-ILZE gel electrolyte is shown in Figure 1c. In this figure, many fibers can be seen, which are interconnected and interwoven into a 3D network. Hence, when the Zn^2+^ ions around the tips are depleted, there are not enough ions to replenish rapidly, and then the growth of tips will slow down or even terminate [52]. As a result, large Zn plate aggregations caused by free tip growth can be largely suppressed, resulting in a uniform and dendrite-free Zn plating [53]. This phenomenon is also consistent with Figure 4d–g. Figure 4e summarizes the interfacial morphology change at the surface of the electrode in the ILZE and BLO-ILZE electrolytes described above, showing that the Zn electrode has a stable SEI layer after cycling in BLO-ILZE. The composition of the Zn surfaces after cycling is analyzed by energy dispersive spectrometry (EDS) to further verify their homogeneity (Figure 4i–k and Figures S7–S9). As shown in Figures S7–S9, EDS analysis shows the SEI layers in the Zn(BF_4_)_2_ aqueous electrolyte, ILZE, and BLO-ILZE mainly consist of Zn and F elements. It is obvious that after cycling with the BLO-ILZE electrolyte, the content and distribution of the elements (Zn, F) are much more concentrated and homogeneous (Figure 4h–j). This can demonstrate the uniform deposition of Zn^2+^. The Zn surface after cycling with the Zn(BF_4_)_2_ aqueous electrolyte is much rougher than with ILZE and BLO-ILZE. And the results of EDS can show that the by-products produced by the cells after cycling are zinc-fluorine compounds, and these products constitute the dense SEI layer [54]. Accordingly, it can be considered that the usage of the BLO-ILZE electrolyte can notably facilitate uniform Zn deposition, avoid side reaction and guarantee the reversible cycling of Zn as discussed above. The ILZE and BLO-ILZE electrolytes have a low water content, so their HER reactions are reduced. And a more uniform SEI layer is generated, so the battery can cycle more stably [55]. This result is further verified by laser microscopy, which can show the surface topography of the zinc anode. After cycling in the ILZE electrolyte, the surface of the Zn foil became rough, with a large number of dendrites at 75.476 μm (Figure 4k). This irregular dendrite growth may puncture the separator and cause a short circuit in the cell [56]. In contrast, in the BLO-ILZE gel electrolyte, the circulating zinc foil surface is very smooth, with a maximum height of 18.457 μm (Figure 4l). Therefore, the ILZE electrolyte and BLO-ILZE electrolyte are selected for follow-up research.

### 3.4. Electrochemical Performance of Zn/Cu Asymmetric Cells

**Figure 5:**
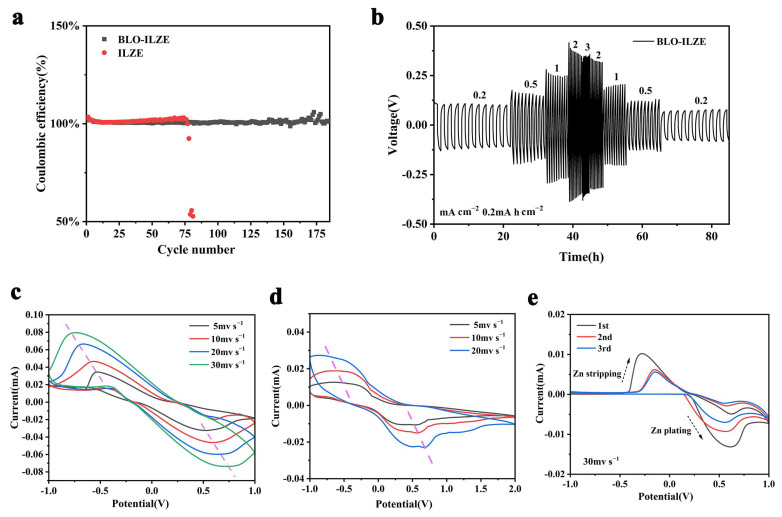
(**a**) CE comparison of Zn/Cu asymmetric cells with ILZE and BLO-ILZE electrolyte. (**b**) The rate performance of Zn/Zn cells with BLO-ILZE electrolyte. (**c**) CV curves of the Zn/ILZE/Zn cell at different scan rates. (**d**) CV curves of the Zn/BLO-ILZE/Zn cell at different scan rates. (**e**) CV curves of the Zn/BLO-ILZE/Zn cell at a scan rate of 30 mv s^−1^.

In order to further understand the reversibility of Zn plating/stripping chemistry, Zn/Cu asymmetric cells with ILZE and BLO-ILZE as electrolytes are assembled and their electrochemical behaviors are measured. Similar results can be obtained for the Zn/Cu asymmetric cell based on electrolytes at 1 mA cm^−2^. As shown in Figure 5, the Zn/Cu cell assembled with the BLO-ILZE electrolyte is stable for more than 185 cycles, along with a remarkable average CE of about 100%. The polarization voltage remains almost unchanged and stable throughout the cycle. This result indicates that the by-products are hardly formed on the electrode surface. The cells assembled with ILZE cause a short circuit after fewer than 75 cycles. The results suggest that the BLO-ILZE electrolyte can obviously inhibit the side reactions and the formation of detrimental Zn dendrite during zinc deposition/exfoliation in Zn/Cu asymmetric cell cycling.

To further study the effect of hydrogel electrolytes on electrochemical durability, the symmetrical cells are subjected to constant current charging/discharging tests with different current densities (0.2 mA cm^−2^, 0.5 mA cm^−2^, 1 mA cm^−2^, 2 mA cm^−2^, 3 mA cm^−2^, 0.2 mA cm^−2^). As exhibited in Figure 5, BLO-ILZE has a superior rate performance and the polarization voltage is very stable. When the current density decreases, the polarization curve has good repeatability with the same current density as before. The better cycling stability of cells with BLO-ILZE at different current densities demonstrates the positive role of the BLO-ILZE electrolyte, which should be associated with the 3D network of the BLO-ILZE supramolecular gel.

Figure 5c shows the cyclic voltammetry (CV) curves of Zn plating/stripping in the ILZE electrolyte at different scan rates, in which the redox couples are associated with the deposition and dissolution of Zn. The interactions formed between the F and the Zn anode surface are superior charge conduction mediators [57]. After the addition of a potential, the larger current response and smaller potential separation between plating and stripping depict faster kinetics of the redox reactions and better reversibility [58]. Figure 5d shows the cyclic voltammetry (CV) curves of Zn plating/stripping in the BLO-ILZE electrolyte at different scan rates, in which the redox couples are associated with the deposition and dissolution of Zn. It can be seen that with the increase in the sweeping voltage, the oxidation peak gradually shifts to the left and the reduction peak gradually shifts to the right. Figure 5e shows the cyclic voltammograms of the Zn||Zn cell assembled with the BLO-ILZE electrolyte at a scan rate of 30 mV s^−1^. The peaks at about 0.56 V correspond to the stripping of Zn, and the peaks at about −0.13 V correspond to the plating of Zn. The results show obvious redox peaks. The curve shapes are highly coincident, which indicate that the cell assembled with the BLO-ILZE electrolyte has good electrochemical reversibility.

### 3.5. Electrochemical Performance of Full Cells

**Figure 6:**
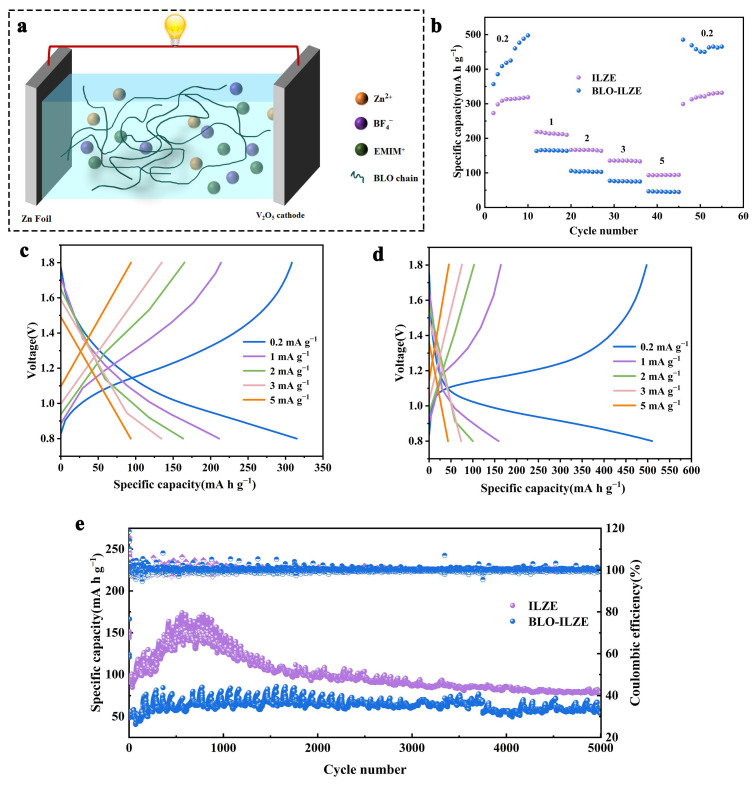
(**a**) Schematic of the V_2_O_5_/BLO-ILZE/Zn full cell. (**b**) Rate performance of ILZE and BLO-ILZE electrolytes. (**c**) GCD curves of the V_2_O_5_/ILZE/Zn full cell at different current densities. (**d**) GCD curves of the V_2_O_5_/BLO-ILZE/Zn full cell at different current densities. (**e**) The long-term current cycling performance of the V_2_O_5_/BLO-ILZE/Zn full cell at 0.5 mA g^−1^.

Figure 6a investigates the Zn-ion storage performance of V_2_O_5_. The full cells were composed of V_2_O_5_/ILZE/Zn and V_2_O_5_/BLO-ILZE/Zn. ZIBs are constructed based on V_2_O_5_ Anodes (Guangdong Canrd New Energy Technology Co., Ltd. Guangdong, China), Zn foil anodes and ILZE electrolyte or the BLO-ILZE electrolyte. The rate performances of two different electrolytes are tested. Figure 6b shows that the BLO-ILZE electrolyte has better multiplying performance. The initial capacity increase indicated an electrochemical activation process as revealed by the gradual decrease in charge-transfer resistance on cycling [59]. The full cell with the BLO-ILZE electrolyte achieves reversible specific capacities of 488, 165.8, 106, 77.2, and 46.6 mA h g^−1^ at current densities of 0.2, 1, 2, 3, and 5 A g^−1^, respectively. Under a low current density, the button cell assembled with the BLO-ILZE electrolyte presents higher current intensity than that of the ILZE electrolyte, indicating higher zinc storage capacity and higher electrochemical reactivity. This may be due to the fast transport kinetics of Zn ions in V_2_O_5_/BLO-ILZE/Zn cells at the anode/electrolyte interface at low current densities [60]. As shown in Figure 6c,d, the full cells of V_2_O_5_/ILZE/Zn and V_2_O_5_/BLO-ILZE/Zn exhibit excellent rate performance and cycling stability from 0.2 to 5 mA g^−1^. The specific capacities of the two cells are very close to each other at a low cycling rate, while the difference in capacity becomes obvious when the current density at 0.2 mA g^−1^. As shown in Figure 6d, the V_2_O_5_/BLO-ILZE/Zn full cell exhibits a reversible capacity of 488 mA h g^−1^ at 0.2 mA g^−1^. The GCD curve shows the oxidation/reduction plateau for the redox conversion of vanadium due to the co-insertion/extraction of Zn^2+^, which corresponds to the CV curve [59,60,61].

As shown in Figure 6e, the long-term current cycling performance of the Zn/V_2_O_5_ full cell using different electrolytes at 0.5 A g^−1^ is measured. It can be seen from Figure 6e that with the progress of the cycle, the capacity of the full cell using ILZE begins to decline. After 800 cycles, the capacity drops from 170 mA h g^−1^ at the beginning to less than 100 mA h g^−1^. However, the capacity of the full cell with the BLO-ILZE electrolyte can remain at 80 mA h g^−1^ and the average coulombic efficiency reaches 100%. Comparing with V_2_O_5_/ILZE/Zn, Zn^2+^ in the V_2_O_5_/BLO-ILZE/Zn full cell can be peeled off/plated in a highly reversible manner and the cycling stability is greatly improved. Dinglei Geng et al. [61] found that the capacity retention of MnO_2_/ZnSO_4_/Zn full battery retains 47% after 800 cycles of charge/discharge at 0.5 A g^–1^. Therefore, the cycling stability of V_2_O_5_/ILZE/Zn and V_2_O_5_/BLO-ILZE/Zn is higher than that of the commonly used the ZnSO_4_ aqueous electrolyte. As mentioned above, because BLO-ILZE can inhibit the formation of Zn dendrite and generate a uniform and dense SEI layer, it can improve the stability of the full cell. The V_2_O_5_/BLO-ILZE/Zn full cell demonstrates high cycling stability with a capacity retention of 99.56% after 5000 cycles.

## 4. Conclusions

In summary, this study reports on a supramolecular gel IL electrolyte (BLO-ILZE) for zinc-ion batteries. A class of highly conductive ionic gel electrolyte was prepared by simple and efficient procedure involving gelation of imidazolium IL EMIMBF_4_ and Zn(BF_4_)_2_ by a novel oxalamide gelator. The supramolecule gel electrolyte can self-assemble on the separator in situ, directly yielding a usable gel electrolyte. Unlike traditional chemically crosslinked polymer gel electrolytes, the supramolecular gel electrolyte can reversibly transform between gel and liquid states, thus facilitating the gel-involved battery assembly and recycling. Furthermore, this BLO-ILZE electrolyte can avoid the hydrogen evolution that occurs in aqueous systems and restrains the growth of zinc dendrite on the electrode. The conductivity of this electrolyte was tested at −70~100 °C, and the conductivity of the supramolecular gel electrolyte was still good at low temperatures. The conductivity of the supramolecular gel electrolyte can reach 2.12 mS cm^−1^ at −40 °C. This is equivalent to the conductivity of aqueous zinc-ion batteries (ZnSO_4_/H_2_O) at −10 °C. We achieved dendrite-free Zn plating/stripping over 3470 hours at 1 mA cm^−2^, which is approximately 860 cycles, with a coulombic efficiency of about 100%. The cycle time of symmetrical cells with the BLO-ILZE electrolyte was 8.5 times higher than that of aqueous zinc-ion batteries. HER and corrosion of Zn are substantially avoided. The symmetric Zn/Zn cell with the BLO-ILZE electrolyte had better cycling stability than the ILZE electrolyte, indicating that the inhibition effect on zinc dendrite is remarkable. A full Zn-ion battery adopting the developed electrolyte delivers over 5000 cycles with 98% capacity at about 100% coulombic efficiency. Meanwhile, it can operate within a wide temperature window. This BLO-ILZE supramolecular gel electrolyte was expected to be applied in the field of energy storage and solve problems in extreme environments.

## Data Availability

Data are contained within the article.

## Supplementary Materials

The following supporting information can be downloaded at: https://www.mdpi.com/article/10.3390/polym16121680/s1. Figure S1: Electrochemical impedance plots of ILZE electrolyte at different temperature. Figure S2: Electrochemical impedance plots of BLO-ILZE electrolyte at different temperature. Figure S3: DSC diagram of 2M Zn(BF_4_)_2_, ILZE, and different concentrations of BLO-ILZE supramolecular gel electrolyte at 25 °C. Figure S4: DSC diagram of 2M Zn(BF_4_)_2_, EMIMBF_4_, and ILZE. Figure S5: Voltage profiles of Zn/Zn symmetrical batteries with different electrolyte at the current density of 0.5 mA cm^−2^ and 5 mA cm^−2^. Figure S6: Voltage profiles of Zn/Zn symmetrical batteries with different concentration of BLO-ILZE electrolyte at the current density of 0.5 mA cm^−2^, 1 mA cm^−2^ and 2 mA cm^−2^. Figure S7: EDS spectra of elements of the Zn electrode after 20 cycles with the Zn(BF_4_)_2_ aqueous electrolyte. Figure S8: EDS spectra of elements of the Zn electrode after 20 cycles with ILZE electrolyte. Figure S9: EDS spectra of elements of the Zn electrode after 20 cycles with BLO-ILZE electrolyte. Figure S10: The schematic image of interfacial change at the interface of electrode/electrolyte. Table S1: The positions corresponding to different peaks of ILZE electrolyte. Table S2: The positions corresponding to different peaks of BLO gelator. Table S3: The cycle life of Zn/Zn symmetrical batteries with different electrolytes.
