# Supplementary material for: An Ionic Liquid Supramolecular Gel Electrolyte with Unique Wide Operating Temperature Range Properties for Zinc-Ion Batteries
Source: Polymers (Basel). 2024 Jun 13;16(12):1680. doi: 10.3390/polym16121680 (PMC11207442; doi:10.3390/polym16121680)
Supplement: Supplementary file 1 [file polymers-16-01680-s001.zip › polymers-3038256-supplementary materials.pdf]

polymers

MDPI

Supporting Information

An ionic liquid supramolecular gel electrolyte with unique wide temperature properties for zinc-ion batteries

Hui Li, Changmiao Huang, Zixuan Teng, Yushu Luo, Chaocan Zhang, Lili Wu, Wenchao Huang, Tingting Zhao, Lijie Dong and Wanyu Chen *

School of Materials Science and Engineering, Wuhan University of Technology, Wuhan 430070, China; lh370765288@whut.edu.cn (H.L.); 317428@whut.edu.cn (C.H.); 281463@whut.edu.cn (Z.T.); 281387@whut.edu.cn (Y.L.); polymers@whut.edu.cn (C.Z.); polym_wl@whut.edu.cn (L.W.); wenchao.huang@whut.edu.cn (W.H.); zhaotingting@whut.edu.cn (T.Z.); dong@whut.edu.cn (L.D.)

* Correspondence: chenwanyu@whut.edu.cn

Supporting Figure

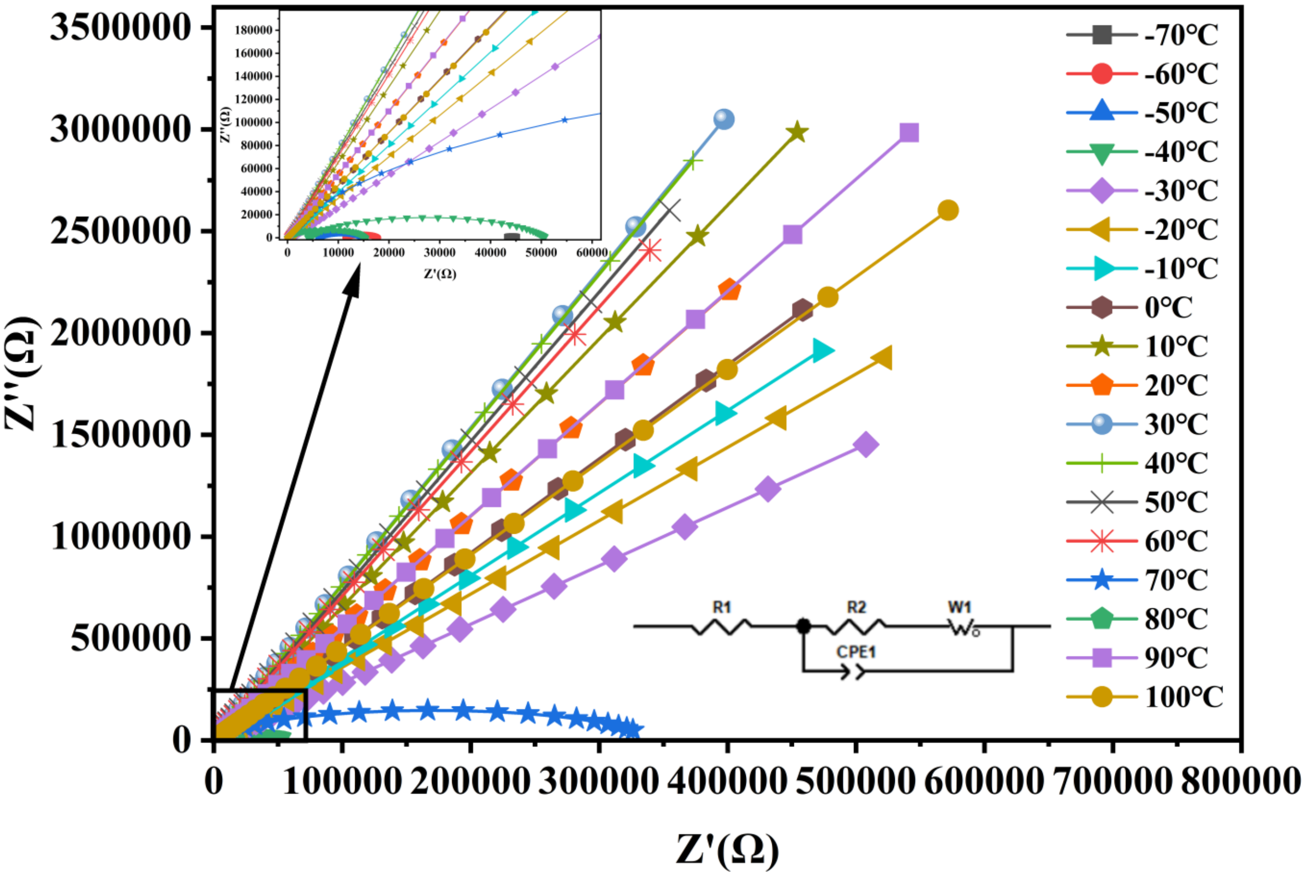

Figure S1. Electrochemical impedance plots of ILZE electrolyte at different temperature.

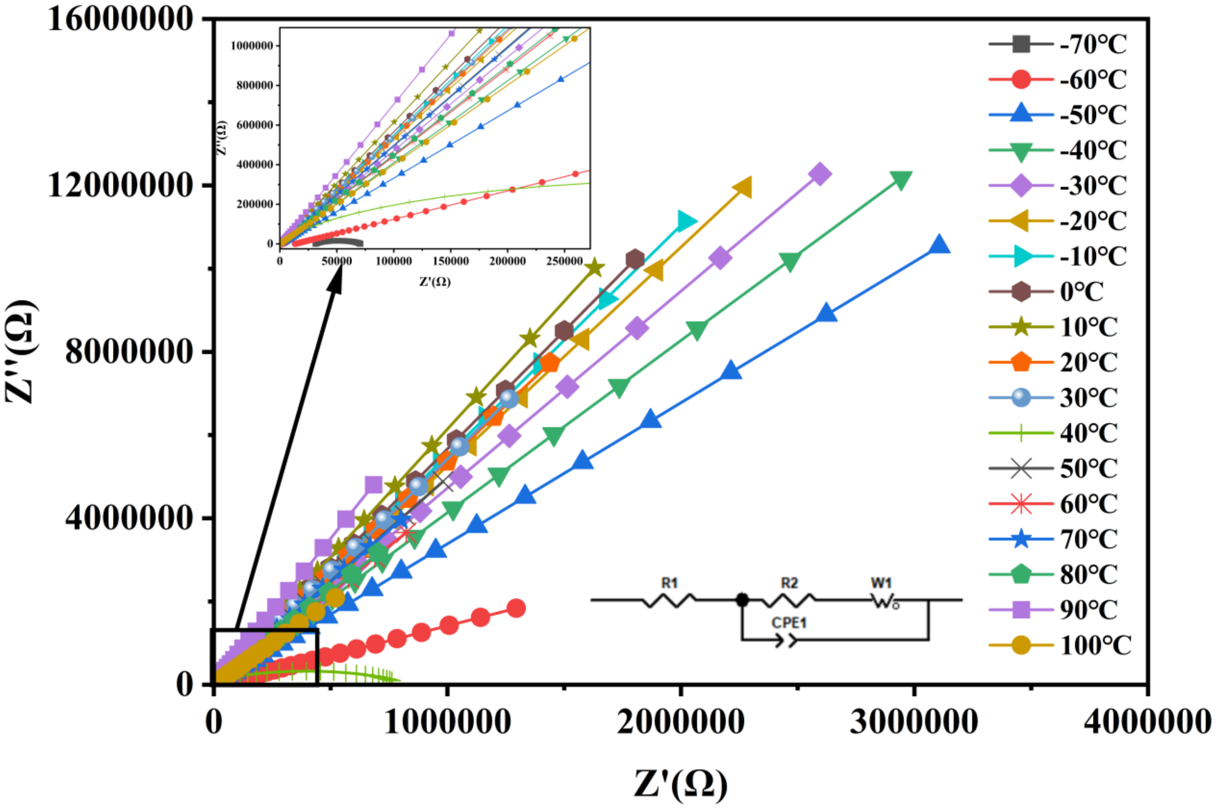

Figure S2. Electrochemical impedance plots of BLO-ILZE electrolyte at different temperature.

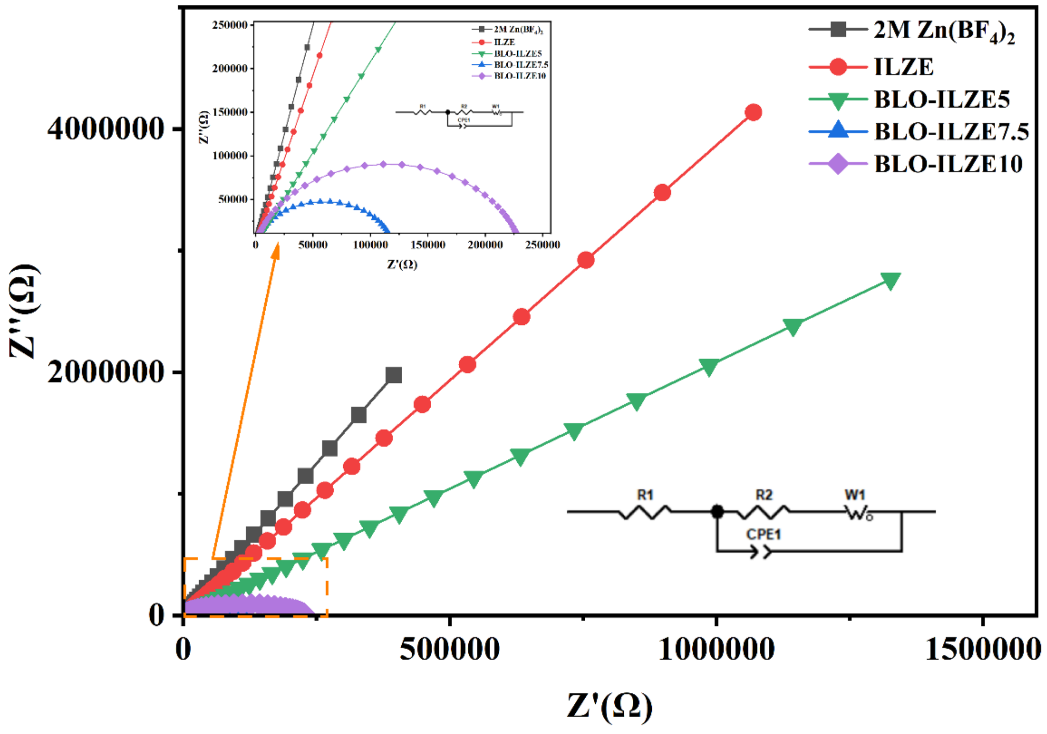

Figure S3. DSC diagram of 2M $Zn(BF_4)_2$, ILZE and different concentrations of BLO-ILZE supramolecular gel electrolyte at 25°C.

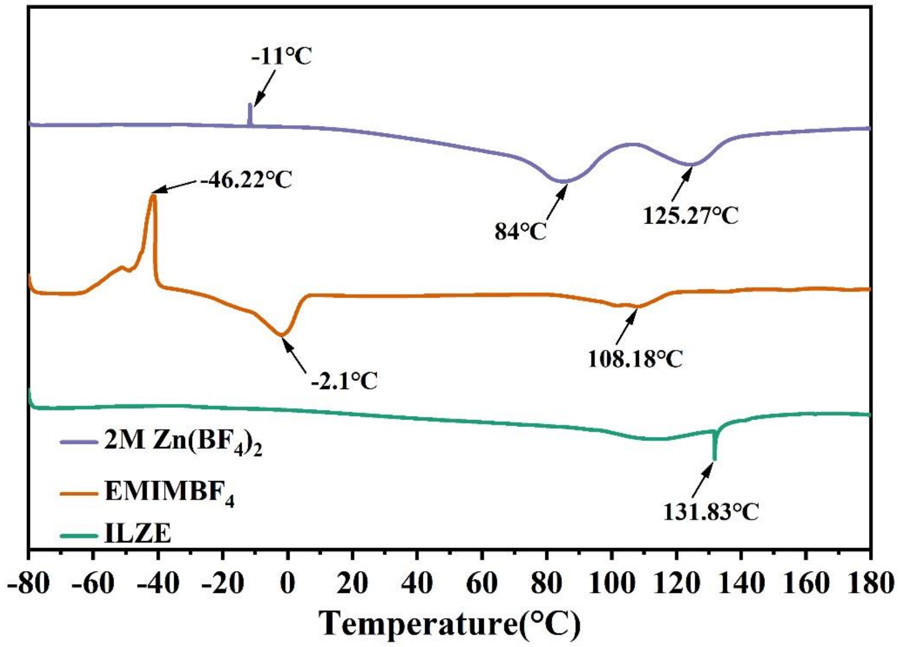

Figure S4. DSC diagram of 2M $Zn(BF_4)_2$, $EMIMBF_4$ and ILZE.

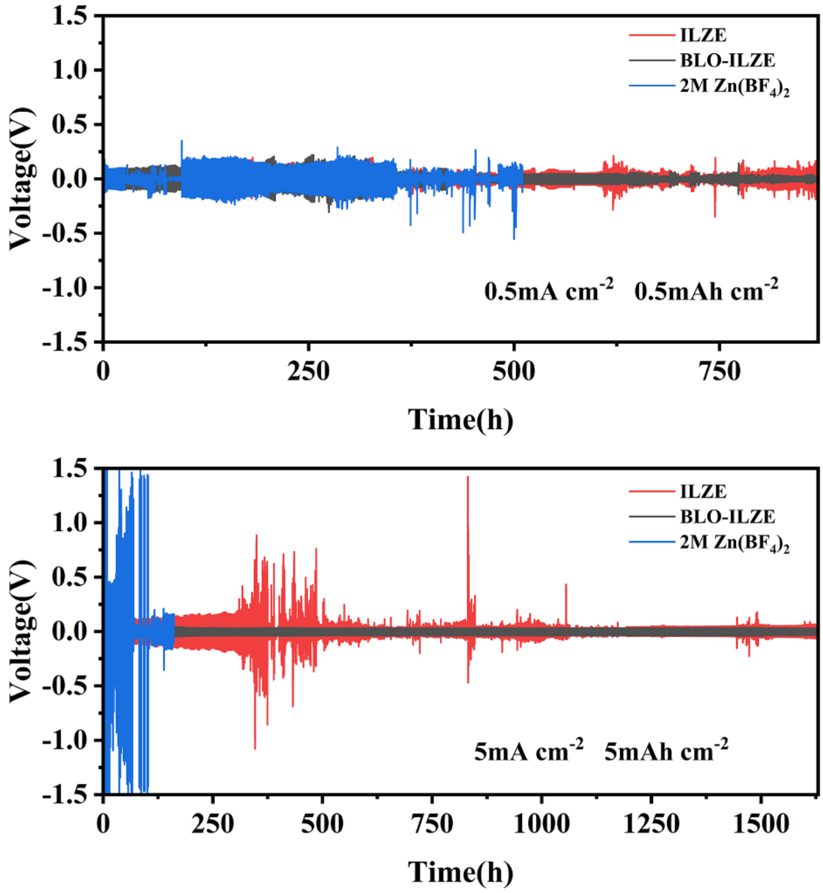

Figure S5. Voltage profiles of Zn/ Zn symmetrical batteries with different electrolyte at the current density of 0.5mA cm^{-2} and 5mA cm^{-2}.

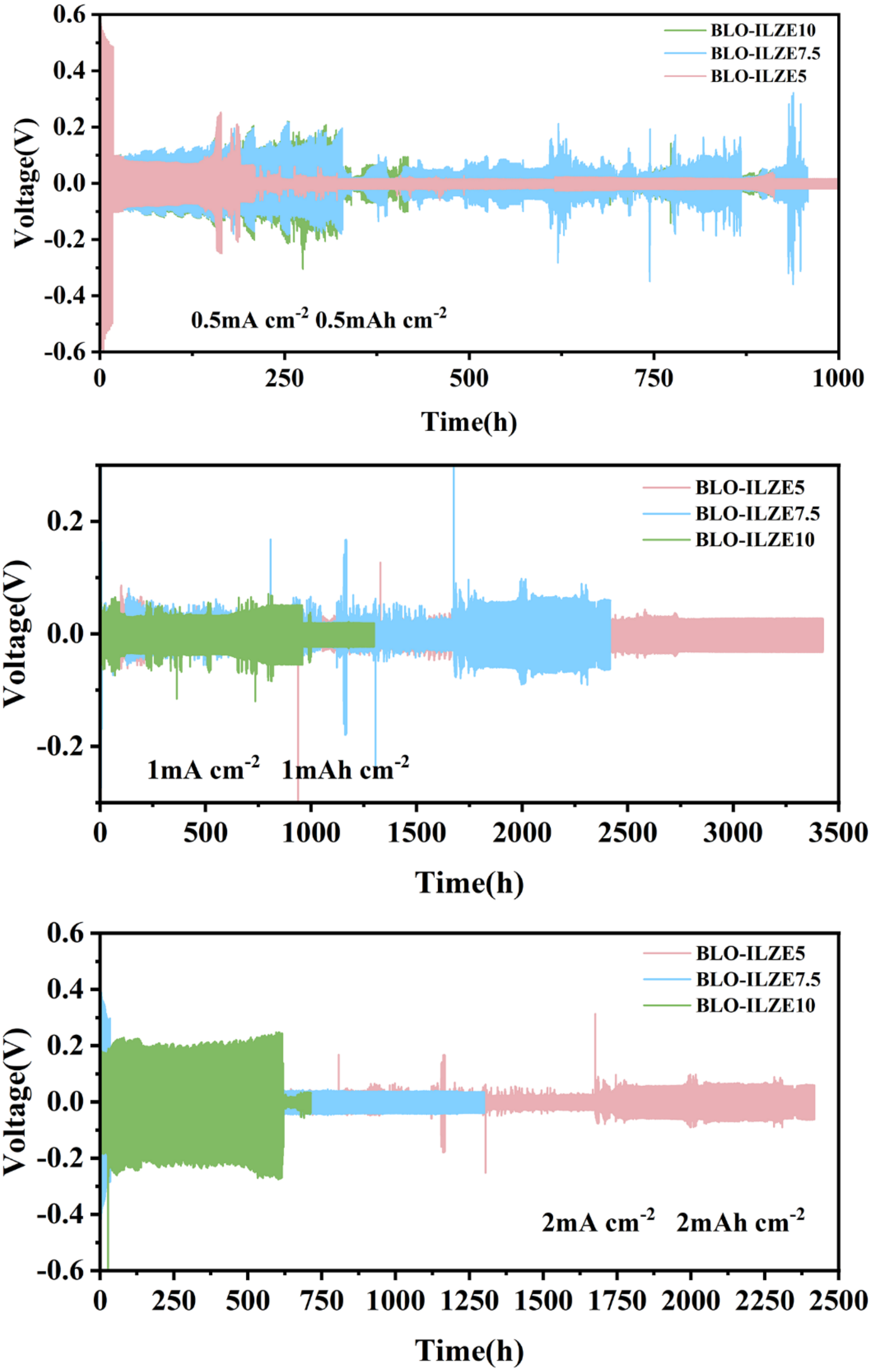

Figure S6. Voltage profiles of Zn/ Zn symmetrical batteries with different concentration of BLO-ILZE electrolyte at the current density of 0.5mA cm^{-2}, 1mA cm^{-2} and 2mA cm^{-2}.

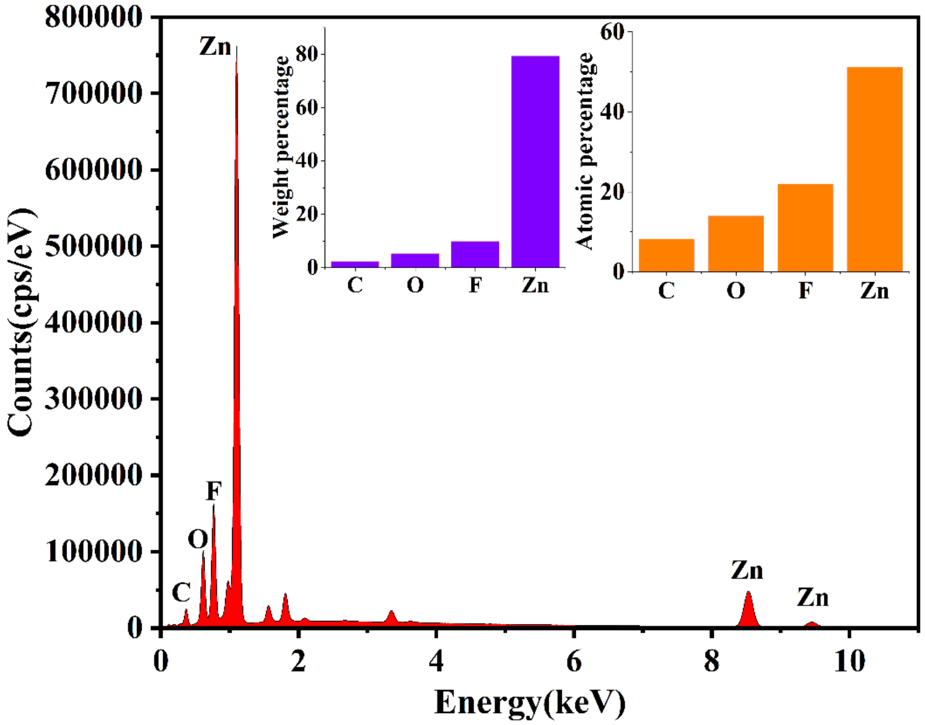

Figure S7. EDS spectra of elements of the Zn electrode after 20 cycles with the $Zn(BF_4)_2$ aqueous electrolyte.

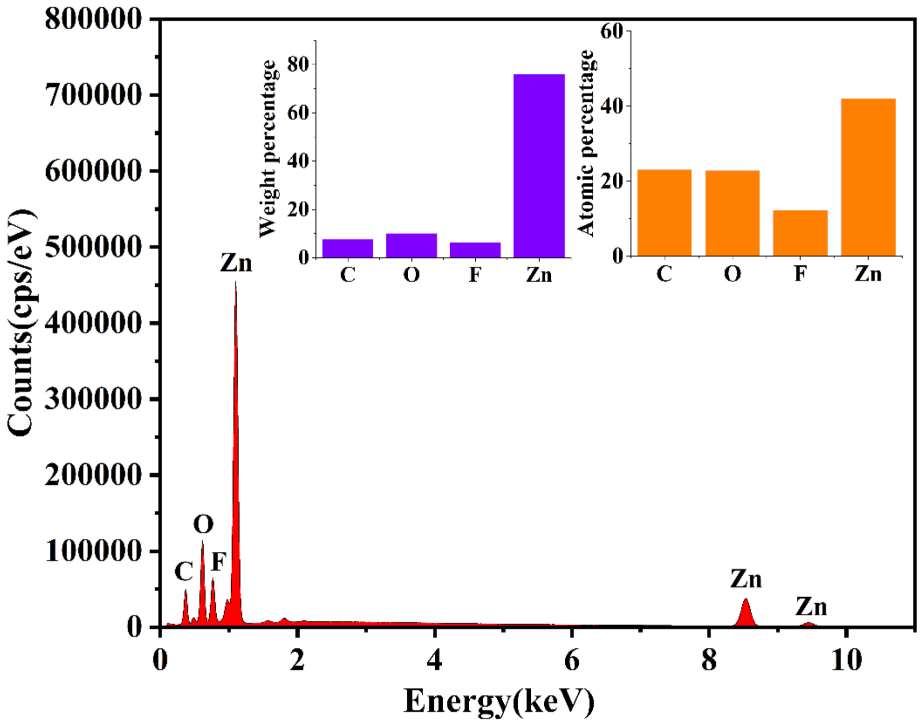

Figure S8. EDS spectra of elements of the Zn electrode after 20 cycles with ILZE electrolyte.

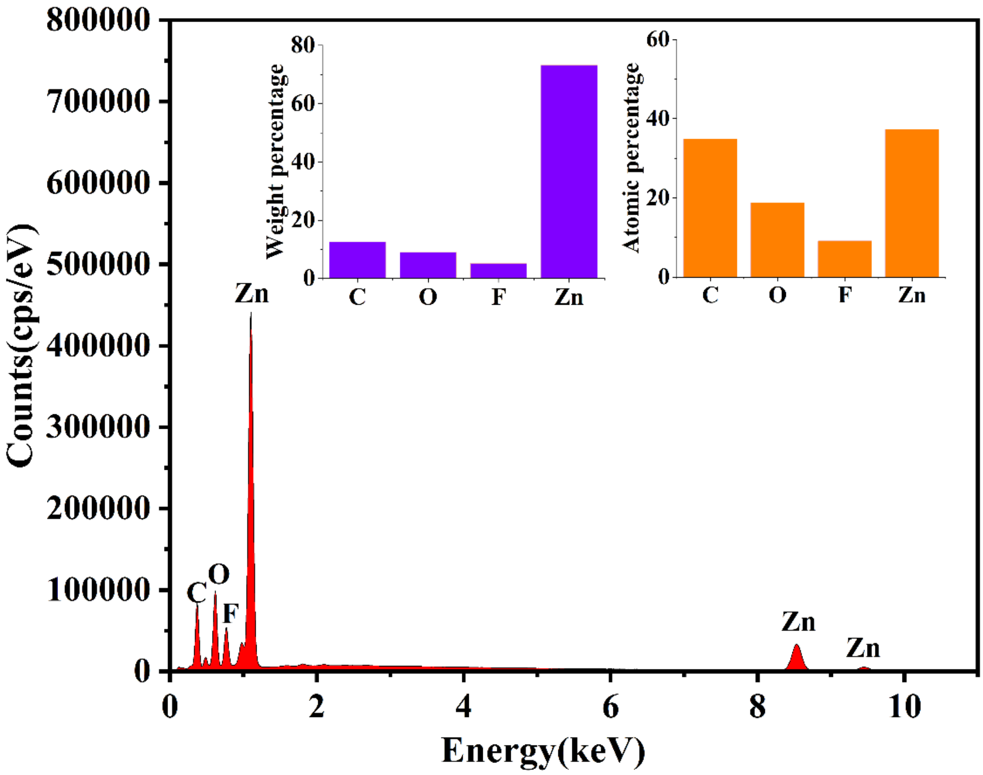

Figure S9. EDS spectra of elements of the Zn electrode after 20 cycles with BLO-ILZE electrolyte.

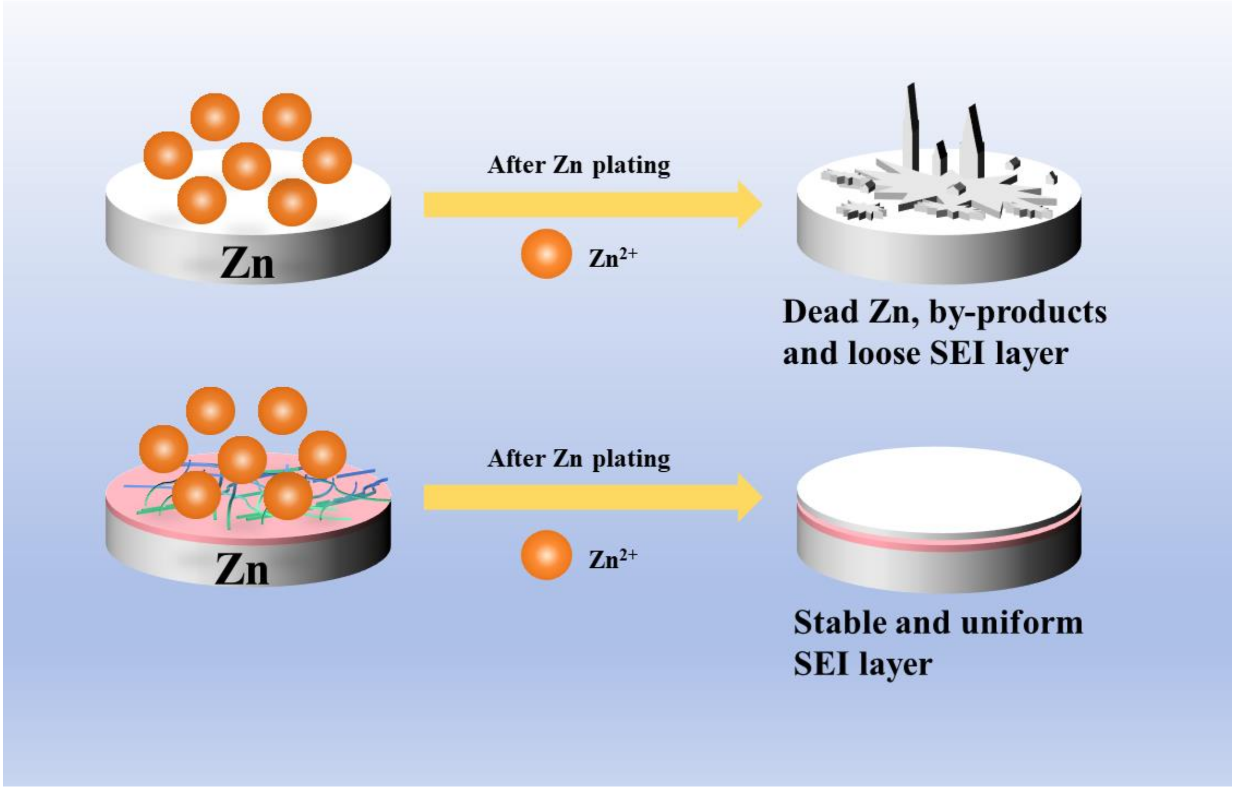

Figure S10. The schematic image of interfacial change at the interface of electrode/electrolyte.

Supporting Table

Table S1. The positions corresponding to different peaks of ILZE electrolyte.

Type of group (v)	wavenumber (cm^{-1})
O-H	3542
-C=C-H	3000
C=O	1636
$C=N^+$	1575

Table S2. The positions corresponding to different peaks of BLO gelator.

Type of group (v)	wavenumber (cm^{-1})
O-H	3378
N-H	3284
C-H	2956, 2874
C=O	1654

Table S3. The cycle life of Zn/ Zn-symmetric batteries with different electrolytes.

Electrolyte	Density/Capacity (mA cm^{-2}/mA h cm^{-2})	Lifespan (hours)	references
2M $Zn(BF_4)_2$/ H_2O	1/1	325	This work
ILZE	1/1	1100	
BLO-ILZE	1/1	3470	
$ZnSO_4$/ H_2O	1/1	400	62]
$Zn(BF_4)_2$/DMC/EC	1/1	＞1230	[63]
$Zn(BF_4)_2$/DMC	1/1	50	[63]
ZnBF/VC	1/1	1000	[64]
ZnBF/H_2O	1/1	90	[64]
4 M $Zn(BF_4)_2$/ H_2O	0.5/0.5	＞250	[65]
Peptide Gel Electrolytes	1/1	1500	[66]
gum arabic/$ZnSO_4$/ Li_2SO_4	0.2/0.2	> 1300	[67]

MD modeling process: Firstly, the ionic models of Zn^{2+}, BF_4^-, $EMIM^+$ and the molecular models of H_2O and BLO were constructed using Materials Studio. Parameters were set through CASTEP module and DMol3 module. Set up the force field and the charge. Molar ratios of the components in the BLO-ILZE electrolyte were calculated. The cubic box of the BLO-ILZE electrolyte was then constructed by the Amorphous Cell module program. The COMPASSII force field was used to calculate the interactions between the components in the Forcite module. The model was first geometrically optimized by setting the number of iterations to 5000 and running at 10 cores. To further equilibrate the model, structural relaxation was performed, and a short energy minimization geometric optimization was performed to optimize the model. The system was subjected to 500 repeated annealing cycles from 300K to 400K. Using the NPT system, that is, the lineages with standard particle number (N), pressure (P), and temperature (T) were subjected to 2000 steps of molecular dynamics simulations at a constant pressure of 1bar with a time step of 1fs. Temperature and pressure were controlled using a Nose thermostat and Berendsen barostat, respectively.

Reference

62. Yang, Y.; Huang, C.; Li, H.; Teng, Z.; Zhang, H.; Wei, X.; Zhang, H.; Wu, L.; Zhang, C.; Chen, W. Study of a Novel Supramolecular Hydrogel Electrolyte for Aqueous Zinc Ion Batteries. *J Mater Chem C* **2023**, *11*, 9559–9569, doi:10.1039/D3TC01284K.
63. He, R.; Yu, F.; Wu, K.; Liu, H.-X.; Li, Z.; Liu, H.K.; Dou, S.-X.; Wu, C. A Dual Organic Solvent Zn-Ion Electrolyte Enables Highly Stable Zn Metal Batteries. *Nano Lett* **2023**, *23*, 6050–6058, doi:10.1021/acs.nanolett.3c01406.
64. Wang, S.; Ying, Y.; Chen, S.; Wang, H.; Cheung, K.K.K.; Peng, C.; Huang, H.; Ma, L.; Zapien, J.A. Highly Reversible Zinc Metal Anode Enabled by Zinc Fluoroborate Salt-Based Hydrous Organic Electrolyte. *Energy Storage Mater* **2023**, *63*, 102971, doi:10.1016/j.ensm.2023.102971.
65. Sun, T.; Yuan, X.; Wang, K.; Zheng, S.; Shi, J.; Zhang, Q.; Cai, W.; Liang, J.; Tao, Z. An Ultralow-Temperature Aqueous Zinc-Ion Battery. *J Mater Chem A* **2021**, *9*, 7042–7047, doi:10.1039/D0TA12409E.
66. Wang, Y.; Liu, X.; Ge, R.; Moretti, M.; Yin, J.; Zhao, Z.; Valle-Pérez, A.U.; Liu, H.; Tian, Z.; Guo, T.; et al. Peptide Gel Electrolytes for Stabilized Zn Metal Anodes. *Acs Nano* **2024**, *18*, 164–177, doi:10.1021/acsnano.3c04414.
67. Wu, K.; Cui, J.; Yi, J.; Liu, X.; Ning, F.; Liu, Y.; Zhang, J. Biodegradable Gel Electrolyte Suppressing Water-Induced Issues for Long-Life Zinc Metal Anodes. *Acs Appl Mater Inter* **2022**, *14*, 34612–34619, doi:10.1021/acsami.2c05887.

Disclaimer/Publisher's Note: The statements, opinions and data contained in all publications are solely those of the individual author(s) and contributor(s) and not of MDPI and/or the editor(s). MDPI and/or the editor(s) disclaim responsibility for any injury to people or property resulting from any ideas, methods, instructions or products referred to in the content.
